# Prognostic Risk Assessment and Prediction of Radiotherapy Benefit for Women with Ductal Carcinoma In Situ (DCIS) of the Breast, in a Randomized Clinical Trial (SweDCIS)

**DOI:** 10.3390/cancers13236103

**Published:** 2021-12-03

**Authors:** Fredrik Wärnberg, Per Karlsson, Erik Holmberg, Kerstin Sandelin, Pat W. Whitworth, Jess Savala, Todd Barry, Glen Leesman, Steven P. Linke, Steven C. Shivers, Frank Vicini, Chirag Shah, Sheila Weinmann, Gregory Bruce Mann, Troy Bremer

**Affiliations:** 1 Department of Surgery, Institute of Clinical Sciences, Sahlgrenska Academy, University of Gothenburg, 405-30 Gothenburg, Sweden; 2 Department of Oncology, Institute of Clinical Sciences, Sahlgrenska Academy, University of Gothenburg, 405-30 Gothenburg, Sweden; per.karlsson@oncology.gu.se (P.K.); erik.holmberg@gu.se (E.H.); 3 Department of Molecular Medicine and Surgery, Karolinska Institutet, 171 77 Stockholm, Sweden; kerstin.sandelin@ki.se; 4 Nashville Breast Center, Nashville, TN 37203, USA; pwhitworth@tmebcn.com; 5 PreludeDx, Laguna Hills, CA 92653, USA; jsavala@preludedx.com (J.S.); glenleesman@gmail.com (G.L.); sshivers@preludedx.com (S.C.S.); 6 Todd S. Barry Dx, Mission Viejo, CA 92691, USA; toddsbarry@icloud.com; 7 Steven P. Linke Consulting, Carlsbad, CA 92009, USA; splinke@gmail.com; 8 Genesis Care, Farmington Hills, MI 48334, USA; frank.vicini2@usa.genesiscare.com; 9 Department of Radiation Oncology, Cleveland Clinic, Cleveland, OH 44195, USA; shahc4@ccf.org; 10 Center for Health Research, Kaiser Permanente Northwest, Portland, OR 97227, USA; sheila.weinmann@kpchr.org; 11 Department of Surgery, University of Melbourne, Melbourne VIC 3010, Australia; bruce.mann@mh.org.au

**Keywords:** DCIS, radiotherapy, prognosis, prediction, risk signature

## Abstract

**Simple Summary:**

Despite clinical advancements in the diagnosis and treatment of DCIS, tailoring individual treatment for women diagnosed with DCIS remains an unmet clinical need. Definitive predictive tools that can predict who will or not benefit from radiation therapy (RT) after breast conserving surgery (BCS) remains elusive. Here, we used a prospective–retrospective design to validate DCISionRT^®^, using data from the SweDCIS randomized clinical trial. DCISionRT identified women with elevated recurrence risk who benefited substantially from RT after BCS. In addition, the test identified women with low recurrence risk and little benefit from RT. These results support our conclusions that knowledge of the individual risk and benefit from RT provided by the test can help clinicians and patients make individualized treatment decisions for women diagnosed with DCIS.

**Abstract:**

Prediction of radiotherapy (RT) benefit after breast-conserving surgery (BCS) for DCIS is crucial. The aim was to validate a biosignature, DCISionRT^®^, in the SweDCIS randomized trial. Women were randomly assigned to RT or not after BCS, between 1987 and 2000. Tumor blocks were collected, and slides were sent to PreludeDx^TM^ for testing. In 504 women with complete data and negative margins, DCISionRT divided 52% women into Elevated (DS > 3) and 48% in Low (DS ≤ 3) Risk groups. In the Elevated Risk group, RT significantly decreased relative 10-year ipsilateral total recurrence (TotBE) and 10-year ipsilateral invasive recurrence (InvBE) rates, HR 0.32 and HR 0.24, with absolute decreases of 15.5% and 9.3%. In the Low Risk group, there were no significant risk differences observed with radiotherapy. Using a cutoff of DS > 3.0, the test was not predictive for RT benefit (*p* = 0.093); however, above DS > 2.8 RT benefit was greater for InvBE (interaction *p* = 0.038). Recurrences at 10 years without radiotherapy increased significantly per 5 DS units (TotBE HR:1.5 and InvBE HR:1.5). Continuous DS was prognostic for TotBE risk although categorical DS did not reach significance. Absolute 10-year TotBE and InvBE risks appear sufficiently different to indicate that DCISionRT can aid physicians in selecting individualized adjuvant DCIS treatment strategies. Further analyses are planned in combined cohorts to increase statistical power.

## 1. Introduction

Four randomized clinical trials (RCT) demonstrated that 30% of patients diagnosed with breast ductal carcinoma in situ (DCIS) recur after breast conserving surgery (BCS) at 10 years [1]. Radiotherapy (RT) after BCS for DCIS has been shown to reduce recurrence rates to 15% at 10 years (50% relative risk reduction) with no effect on mortality [1]. These and subsequent studies investigated clinicopathological factors, biomarkers, or gene expression-based assays that are prognostic for recurrence [2,3,4,5]. Such factors have been used to identify groups with lower local recurrence risk [6]. However, the RT relative risk reduction remains about 50% in all different risk groups.

The DCISionRT^®^ biosignature (PreludeDx, CA, USA) combines biomarkers with clinicopathological factors and reports a continuous Decision Score (DS) on a scale (0–10) with corresponding 10-year risks after BCS with and without RT [7,8]. This biosignature was validated in an independent population-based cohort from Kaiser Permanente, Oregon [7] where it was prognostic, and the RT benefit was larger in the Elevated Risk group.

Here we validated DCISionRT for prognostic risk assessment and for RT benefit in the SweDCIS RCT with long-term follow up [9,10].

## 2. Results

Table 1 shows the clinicopathologic characteristics of all women in SweDCIS compared to subsets with negative margins and complete data. Only 33 patients (3%) received tamoxifen [9,10,11]. There were no statistically significant differences between the validation cohort and all women with negative surgical margins in the SweDCIS RCT (Table 1, Supplemental Table S1). There were no statistically significant differences between women treated with or without RT (*n* = 247 and 257, 49% and 51%), except that slightly more women over 70 years of age received RT in the validation cohort (Supplemental Table S2). The median follow-up time was 17.1 years (1^st^ quartile to 3^rd^ quartile: 15.2 to 20.5 years), which was longer than the 10 years used to assess outcomes in this study. After 10 years, there were 59 DCIS and 31 invasive events in the validation cohort. (Supplemental Table S2).

**Figure 1:**
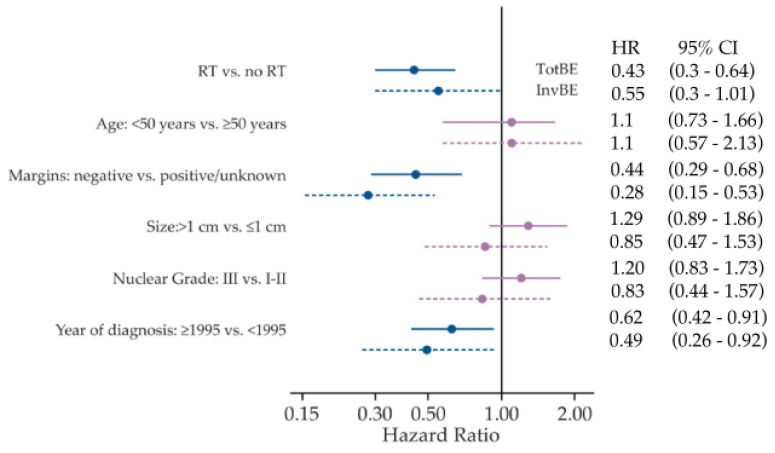
Multivariable analysis in SweDCIS, including patient and tumor characteristics. Hazard ratios with 95% confidence intervals for risk of all ipsilateral breast events (TotBE) and invasive events (InvBE) after 10 years, using the data set with complete biosignature data, including those with positive or unknown margins (*n* = 582).

In women with complete data, multivariable analysis found no statistically significant association between 10-year outcomes and patient age or tumor grade and size after accounting for margin status, RT, and year of diagnosis (Figure 1). Positive margins were associated with higher event rates. RT was associated with approximately 50% lower rates and women diagnosed since 1995 had lower event rates (Figure 1).

In women treated with BCS without RT, relative 10-year event rates increased with increasing continuous DS, with a statistically significant HR of 1.5 per 5 DS units (95% CI 1.1 to 2.3) for TotBE and a HR of 1.5 per 5 DS units (95% CI 0.7 to 2.8) for InvBE. A categorical DS Low Risk group (DS ≤ 3) was pre-specified to identify patients with 10-year 10% TotBE risk and 6% InvBE risk [8]. For DS ≤ 3 versus DS > 3, the HRs for 10-year event rates were 1.5 (95% CI 0.9 to 2.6) for TotBE and 1.7 (95% CI 0.7 to 4.2) for InvBE.

DCISionRT classified approximately half of the women into the Low Risk group (DS ≤ 3, *n* = 240/504, 48%), and the remaining into the Elevated Risk group (DS > 3, *n* = 264/504, 52%). The percentage of women with Low and Elevated Risk within different clinicopathologic factors is reported in Table 2. DCISionRT classified 37% of women with NG 3 or size > 2.5 cm tumors as Low Risk, and 38% of women with NG 1 or 2, and size < 1 cm as Elevated Risk. The test identified 56% of women younger than 50 years as Low Risk, and 40% of women who met “good-risk” criteria from RTOG 9804 [12] as Elevated Risk.

The absolute 10-year rates and effect of RT on 10-year event rates differed by DS Low and Elevated Risk group, as presented in Table 3. RT improved outcomes for patients within the Elevated Risk group, with a statistically significant reduction in absolute TotBE rate of 15.5% (95% CI 5.9% to 25.0%) and InvBE 9.3% (95% CI 2.0% to 16.5%). However, within the Low Risk group, no statistically significant differences were found, with observed differences of 5.7% (95% CI −0.8% to 12.2%) and 1.2% (95% CI −5.7% to 8.2%).

In women treated with BCS without RT, TotBE relative 10-year event rates increased with increasing continuous DS, with a statistically significant HR of 1.5 per 5 DS units (95% CI 1.1–2.3). For InvBE, the relative risk increased, though not statistically significantly, with a HR of 1.5 per 5 DS units (95% CI 0.7–2.8). For categorical DS Risk groups (DS ≤ 3 vs DS > 3), the HRs for 10-year event rates were 1.5 (95% CI 0.9–2.6) for TotBE and 1.7 (95% CI 0.7–4.2) for InvBE. In the Elevated Risk group, there was a statistically significant relative RT benefit with a 10-year TotBE HR of 0.32 (95% CI 0.17 to 0.58) and InvBE HR of 0.24 (95% CI 0.08 to 0.74), see Table 4. In the Low Risk group, the relative RT difference was not statistically significant, with a TotBE HR of 0.53 (95% CI 0.28 to 1.02) and InvBE HR of 0.84 (95% CI 0.30 to 2.31). When using the categorical DS threshold of 3.0, the *p*-values for the multiplicative interactions between RT and DS risk groups were 0.24 for TotBE and 0.093 for InvBE.

According to the predefined statistical analysis plan (SAP) we assessed an optimal threshold for predicting differential relative RT effect by DS. The multiplicative interaction of DS with RT (RT: DS > x) was assessed for DS thresholds between 1.0 and 3.0, Supplemental Table S3. The multiplicative interaction between RT and DS did not vary significantly (*p* > 0.05) depending on DS threshold for TotBE rate but did vary significantly (*p* < 0.05) depending on DS threshold for InvBE rate. There was a statistically significant interaction (*p* = 0.035) for a DS threshold of 2.8 for 10-year InvBE rate. The RT benefit for 10-year InvBE rate was statistically significant above a DS threshold of 2.8 with HR 0.22 (0.07 to 0.68) but was not statistically significant below a DS threshold of 2.8 with HR 1.15 (0.38–3.41).

## 3. Discussion

One key finding from this validation of DCISionRT was that continuous DS was prognostic for 10-year recurrence rates. Also, using the pre-specified DS threshold of 3.0 to define DS Low and Elevated Risk groups, approximately 50% of women were classified into the Elevated Risk group. In the DS Elevated Risk group, the addition of RT reduced absolute 10-year rates for TotBE by 15.9%, and for InvBE by 9.4%. This represented a relative RT benefit of approximately 70% for both DCIS and invasive recurrences, consistent with prior studies [7,8]. This observed RT benefit is higher than the expected 50% relative risk reduction, and RT benefit was greater above DS threshold 2.8 than below for InvBE. Furthermore, the test identified a Low Risk group who had 10-year DCIS rates of 0.7% and 5.2%, and InvBE rates of 6.5% and 7.7%, treated with and without RT. Finally, many women who would be classified into a particular risk group based on classical clinicopathologic factors were re-classified into the opposite risk groups by DS.

Besides being performed in a randomized cohort, with a pre-specified SAP, this study has some additional strengths. Only 3% of the women in this validation received tamoxifen, which makes the influence of endocrine therapy negligible [10]. Tamoxifen was not routinely used according to Swedish guidelines.

Our results are consistent with prior validations of DCISionRT, including the independent validation by Kaiser Permanente and the cross-validation in cohorts from Uppsala University and the University of Massachusetts [7,8]. In particular, the 10-year InvBE rates in the Low Risk group with and without RT had small differences in the present study (1.2%), and in prior studies (1% to 2%) [7,8]. For women in the Elevated Risk group the relative risk reduction was approximately 70% for both TotBE and InvBE rates, consistent with earlier validations. In our study, relative RT benefit for invasive recurrences was greater for patients with elevated DS results (DS > 2.8), also noted in prior validations. The corresponding 10-year absolute RT benefit (DS > 3) was also consistent with earlier validations [7,8]. This highlights the utility of DCISionRT to aid physicians in identifying women with low 10-year recurrence risk who would derive little benefit from RT, as well as women with elevated risk who would gain substantial RT benefit.

Clinicopathological factors have been explored to identify low-risk patients with limited benefit from RT. However, in randomized trials, including SweDCIS, the relative risk reduction by RT has been about 50% or more in all risk groups [1,13,14]. In RTOG 9804, similar low-risk groups were randomly assigned to RT or not and the study found that omission of RT was associated with increased rates of local recurrence (11% vs. 3%) at 12 years [15]. Together, these studies demonstrate that traditional clinicopathologic features are insufficient to consistently identify low risk DCIS patients without a clinically relevant RT benefit. A gene expression assay has been shown to be prognostic for recurrence risk, but all groups had similar relative RT benefit [4]. In contrast, this validation demonstrated the ability of DCISionRT to identify a low-risk group with a marginal difference from RT of 1.2% for invasive cancer after 10 years, for whom no statistically significant association was found between RT and 10-year event rates. Further, there was a multiplicative interaction for RT InvBE benefit with DS for a DS threshold of 2.8.

While depending on local practices, RT may not be recommended for women with “low-risk” DCIS. However, 30–40% of these women with “low-risk” clinicopathology were re-classified into the DS Elevated Risk group. DCISionRT’s biologic approach and non-linear algorithm may be an explanation why the test is not only prognostic, but also seems to provide predictive value. DCISionRT may aid physicians and patients to make individualized treatment decisions, as exemplified by a 42% change in RT recommendations in an ongoing prospective registry study [16].

A limitation of the present validation is that SweDCIS may not be representative of modern practice. Women diagnosed after 1995 are expected to have lower risks profiles, which was adjusted for in multivariable analysis. Whether or not women were included may have depended on clinicopathological factors or changes in mammographic screening over time. However, 79% of all DCIS cases in SweDCIS were detected by screening, in line with contemporary data [17], and the distribution of clinicopathologic characteristics in the present validation were consistent with those in a more contemporary cohort [7]. Furthermore, today, a positive margin would result in re-excision, while only 80–89% of all women had negative margins in SweDCIS [10,11]. Therefore, only women with negative margins were included in the present validation.

Another limitation was that fewer patients than in the entire SweDCIS RCT were available for analysis with a corresponding lower number of events, resulting in wider confidence intervals and preventing further subset analyses. However, an analysis including multiple cohorts is planned, to assess test performance within different clinicopathological risk groups, such as data presented at SABCS 2020 within patients meeting RTOG 9804 criteria [18].

## 4. Materials & Methods

### 4.1. Biosignature

The DCISionRT biosignature combines protein expression (PR, HER2, Ki67, FOXA1, p16/INK4A, SIAH2, COX2) by immunohistochemistry with clinicopathologic factors (age, tumor size, margin status, palpability). The continuous DS is calculated using a proprietary non-linear algorithm to account for interactions between factors in order to better interpret complex biological information. The biological signature was parameterized and tested using multiple cross-validations and produced a consensus continuous risk score on a scale from zero to ten, termed Decision Score (DS). A risk threshold was selected using the training datasets in the cross-validated development with the goal of identifying an average 10-year IBE risk of 10% and an IBC risk of 6% or less. Patients with a score greater than the threshold belonged to the Elevated Risk Group. The threshold between the Low and Elevated Groups was scaled to 3, with the Low Risk Group including patients with DS ≤ 3, and the Elevated Risk Group including patients with DS > 3 [7,8].

### 4.2. Study Design

Test performance was evaluated on subjects from the SweDCIS RCT [9,10,11] using a prospective–retrospective design pre-specified in a statistical analysis plan. An ipsilateral invasive event (InvBE) was defined as a local or regional breast event. Metastatic events occurring prior to contralateral invasive cancers were considered as invasive events; however, none were identified. A total breast event (TotBE) was defined as a subsequent ipsilateral DCIS or InvBE. Analyses were based on time from surgery to first event. Censoring occurred at 10 years after diagnosis or death. The validation cohort was women with negative margins who had complete data for validating the test.

The objectives were to assess the association of continuous DS with 10-year recurrence rates in women treated without RT, and to assess the association of RT and 10-year recurrence rates within categorical DS Risk groups, which were prespecified using a DS threshold of 3.0 to identify a low risk group with 10-year risks of 10% TotBE and 6% InvBE [8]. Other planned analyses assessed the multiplicative interaction of RT and DS over a range of DS thresholds to identify an optimal threshold for predicting radiotherapy benefit.

### 4.3. Patients and Sample Preparation

Archived formalin-fixed paraffin-embedded tissue blocks were collected from 26 Swedish pathology departments. H&E slides were used to identify representative blocks. Tissue samples were processed in Sweden, and 4-µm whole sections on slides were sent to PreludeDx. DCISionRT testing was performed by PreludeDx while blinded to patient outcomes. Biomarker expression, nuclear grade (NG), and tumor size were determined after verification of DCIS without invasive disease by board-certified pathologists. PreludeDx transmitted biosignature data and test results that were combined with clinical and outcome data in Sweden. Size was defined according to the following priority based on SweDCIS case report forms: microscopic size followed by macroscopic, mammographic, and size from new sections. Surgical margin was reported as negative, positive, or unknown. Outcomes and clinicopathology were determined using the SweDCIS database. Follow-up was completed prior to 15 December 2012. Our database was created for test validation following REMARK criteria. 

The original SweDCIS RCT included 1046 Swedish women diagnosed from 1987 to 2000 who were randomly assigned to whole breast RT or no RT after BCS [11]. Tumor blocks were available for 873 women (83.5%), although 162 had no representative DCIS or presence of invasive cancer. Of the remaining 711 women, 582 had complete biosignature data. A total of 78 had positive or unknown surgical margins, leaving 504 women with negative margins for the validation cohort, reflecting the contemporary practice of complete removal of DCIS (Supplemental Figure S1). Patient and tumor characteristics for subsets of women included and excluded from analyses are listed in Table 1 and Supplemental Table S1. Compared to results presented at San Antonio Breast Cancer Symposium (SABCS) 2017, two duplicates were removed [9].

### 4.4. Statistical Analyses

Analyses were conducted for 10-year TotBE and InvBE rates. Hazard ratios (HR) for association of RT, year of diagnosis and clinicopathologic factors with 10-year rates were determined by multivariable Cox proportional hazards (CPH) analysis. In the validation cohort, HRs for the association of RT with 10-year rates were determined by CPH within categorical DS Risk groups (DS ≤ 3, DS > 3). HRs for the association of continuous DS with 10-year rates were determined by CPH for women treated without RT. Absolute 10-year rates were calculated by Kaplan–Meier analyses, and rates and differences with 95% confidence intervals (CI) were calculated using Cox proportional models by means of flexible parametric survival model (stpm2) [19] and standardized failure function (standsurv) [20]. Using this method, 10-year absolute rates were adjusted for year of diagnosis (cut-off ≥1995), when significant. In order to determine optimal thresholds for assessing RT benefit, interactions between RT and DS were assessed for continuous DS thresholds between 1.0 and 3.0 using the likelihood ratio, comparing a model including a term for the treatment/risk group interaction with a model including only main effects. Multivariable Cox proportional hazards analysis was performed to assess the effect of clinicopathology factors and treatment on the 10-year TotBE risks.

Clinicopathologic factors were summarized by counts and percentages for patient subsets, and classification of patients by DS Risk group within individual clinicopathologic risk factors or RTOG 9804 criteria [12,15] was summarized by counts and percentage. T-test or Fisher’s exact testing was used to assess differences between subsets. Statistical analyses were performed with Stata/MP 16.1 (SweDCIS Study Group) and shadowed in SAS by an independent statistical analysis group (McCloud Consulting Group). This study was approved by the ethics committee at Umeå University, Sweden, Dnr 2005:118, 2005/118/02 and 2014-230-32M. 

## 5. Conclusions

Radiotherapy benefit was statistically significant in patients with elevated DS but not in patients with low DS by DCISionRT. Continuous DS was prognostic for TotBE risk although categorical DS did not reach significance. Absolute 10-year TotBE and InvBE risks appeared sufficiently different to indicate that DCISionRT can aid physicians in selecting individualized adjuvant DCIS treatment strategies. Further analyses are planned in combined cohorts to increase statistical power.

## Figures and Tables

**Table 1:** Patient clinicopathologic factors, treatment, and events for SweDCIS trial cohort and study subsets.

Factors	SweDCIS Trial Cohort (*n* = 1046)		Validation Cohort Negative Margins (*n* = 504)	
Mean Age, (sd, min-max)	57.0 (9.1, 29–79)		57.6 (9.1, 29–79)	
	
Age group, *n* (%)				
<50	252	(24)	116	(23)
50–69	722	(69)	348	(69)
≥70	72	(7)	40	(8)
Year of diagnosis, *n* (%)				
1987–1994	602	(58)	295	(59)
1995–2000	444	(42)	209	(41)
Mode of detection, *n* (%)				
Screening	823	(79)	414	(82)
Non-screening	220	(21)	89	(18)
Missing	3	(0)	1	(0)
Palpable, *n* (%)				
Yes	236	(23)	110	(22)
No	784	(75)	394	(78)
Missing	26	(2)	-	-
Size, *n* (%)				
≤1 cm	476	(46)	240	(48)
>1 cm	533	(51)	264	(52)
Missing	37	(3)	-	-
Surgical margins, *n* (%)				
Negative	857	(82)	504	(100)
Positive	115	(11)	-	-
Missing	74	(7)	-	-
Nuclear grade, *n* (%)				
1	216	(21)	155	(31)
2	221	(21)	164	(32)
3	272	(26)	185	(37)
Missing	337	(32)	-	-
Radiotherapy, *n* (%)				
Yes	526	(50)	257	(51)
No	520	(50)	247	(49)
Hormonal therapy, *n* (%)				
Yes	33	(3)	17	(3)
No	1013	(97)	487	(97)
First ipsilateral events within 10-years, *n* (%)				
New DCIS	120	(11)	59	(12)
InvBE—Invasive BC	87	(8)	31	(6)
InvBE—Metastases	3	(0)	-	-
Censored—BC death	1	(0)	1	(0)
Censored—other death	57	(5)	30	(6)
Censored at end of follow-up	778	(74)	383	(76)
First contralateral events within 10-years, *n* (%)				
New DCIS	16	(2)	7	(1)
Invasive BC	46	(4)	21	(4)

The complete data subset is comprised of patients with complete biosignature data with and without negative margins. The Validation Cohort is comprised of patients with complete biosignature data and negative margins. Abbreviations: BC = Breast Cancer; InvBE = Invasive breast cancer event risk.

**Table 2:** The distribution of clinicopathologic characteristics of patients treated with breast conserving surgery with negative margins in the Validation Cohort by DCISionRT categorical risk groups.

Factors	DS Elevated Risk Group (DS > 3)		DS Low Risk Group (DS ≤ 3)	
	*n*	%	*n*	%
All	264	52%	240	48%
Age				
<50 years	51	44%	65	56%
≥50 years	213	55%	175	45%
Nuclear grade				
1	65	42%	90	58%
2	74	45%	90	55%
3	125	68%	60	32%
Size				
≤1 cm	111	46%	129	54%
>1 cm	153	58%	111	42%
‘Low/high-risk’ clinicopathology criteria				
NG 1 or 2, Size ≤ 1 cm	63	38%	103	62%
NG 3 or Size > 2.5 cm	84	37%	84	63%
RTOG 9804 criteria *	79	40%	118	60%

* ’Low-risk’ clinicopathologic DCIS criteria modified from RTOG 9804 [12], consisting of screen detected, non-palpable lesions with a size ≤ 2.5 cm, NG 1 or 2 and negative margins. Abbreviations: DS = Decision Score, NG = Nuclear Grade. % Represents distribution of patients in each row.

**Table 3:** The 10-year absolute total and invasive breast event risks for patients treated with BCS with negative margins and either treated with or without adjuvant RT.

	Total Ipsilateral Breast Event Risk (TotBE) at 10 Years Absolute Risk (CI 95%) *		Invasive Breast Cancer Event Risk (InvBE) at 10 Years Absolute Risk (CI 95%) *	
Treatment	Elevated Risk (DS > 3), *n* = 264	Low Risk (DS ≤ 3), *n* = 240	Elevated Risk (DS > 3), *n* = 264	Low Risk (DS ≤ 3), *n* =240
BCS without RT	23.8% (14.8%–36.8%)	12.9% (6.9%–23.5%)	12.4% (7.2%–20.8%)	7.7% (3.9%–14.9%)
BCS plus RT	8.3% (4.5%–15.3%)	7.2% (3.5%–14.6%)	3.1% (1.2%–8.1%)	6.5% (3.2%–13.2%)
Absolute risk difference	15.5% (5.9%–25.0%)	5.7% (−0.8%–12.2%)	9.3% (2.0%–16.5%)	1.2% (−5.7%–8.2%)

Risks are provided within the categorical DS Low (DS ≤ 3) and DS Elevated Risk (DS > 3) groups defined by the DCISionRT biologic signature. * Total 10-year breast event (TotBE) risks adjusted for year of diagnosis, and Invasive 10-year breast event (InvBE) risks are given. Abbreviations: 95% CI = 95% Confidence Interval; DS = Decision Score; RT = Radiotherapy.

**Table 4:** The relative risk effect of radiotherapy on 10-year risks for women diagnosed with DCIS and receiving breast conserving surgery (negative margins) within DS Low and Elevated Risk groups defined using DCISionRT.

Risk Group	Relative Rates for RT Treatment at 10 Years	
	TotBE	InvBE
	HR (CI 95%), *p*-Value *	HR (CI 95%), *p*-Value *
DS Elevated Risk (DS > 3), *n* = 264	0.32 (0.17–0.58), *p* < 0.001	0.24 (0.08–0.74), *p* = 0.013
DS Low Risk (DS ≤ 3), *n* = 240	0.53 (0.28–1.02), *p* = 0.059	0.84 (0.30–2.31), *p* = 0.73

The RT effects are provided as a Hazard Ratio (HR) with 95% confidence intervals within DS risk groups, as determined by Cox proportional hazards analysis. The *p*-value for an interaction of RT effect with DS > 3 versus DS ≤ 3 was *p* = 0.24 for TotBE and *p* = 0.093 for InvBE 10-year rates. * The *p*-values for interaction of RT:(DS > x) for cutoffs between 1.0 and 3.0 are presented in Supplemental Table S3. Abbreviations: 95% CI = 95% Confidence Interval; DS = Decision Score; RT = Radiotherapy; HR = Hazard Ratio; TotBE = Total breast event; InvBE = Invasive breast event.

## Data Availability

Additional data is available in supplementary material.

## Supplementary Materials

The following are available online at https://www.mdpi.com/article/10.3390/cancers13236103/s1, Figure S1: Study Design as per REMARKS guidelines; Table S1: Patient clinicopathologic factors, treatment, and events for SweDCIS trial cohort with negative margins and patients included and not included in the Validation Cohort; Table S2: Patient clinicopathologic factors, treatment, and events for SweDCIS trial cohort with negative margins included in the Validation Cohort with and without radiotherapy (RT); Table S3: Multivariable Cox proportional hazards analysis
