# Supplementary material for: Prognostic Risk Assessment and Prediction of Radiotherapy Benefit for Women with Ductal Carcinoma In Situ (DCIS) of the Breast, in a Randomized Clinical Trial (SweDCIS)
Source: Cancers (Basel). 2021 Dec 3;13(23):6103. doi: 10.3390/cancers13236103 (PMC8657230; doi:10.3390/cancers13236103)
Supplement: Supplementary file 1 [file cancers-13-06103-s001.zip › cancers-1441191-Supplementary.pdf]

Prognostic Risk Assessment and Prediction of Radiotherapy Benefit for Women with Ductal Carcinoma In Situ (DCIS) of the Breast, in a Randomized Clinical Trial (SweDCIS)

Fredrik Wärnberg [1,*], Per Karlsson [2], Erik Holmberg [2], Kerstin Sandelin [3], Pat W. Whitworth [4], Jess Savala [5], Todd Barry [6], Glen Leesman [5], Steven P. Linke [7], Steven C. Shivers [5], Frank Vicini [8], Chirag Shah [9], Sheila Weinmann [10], Gregory Bruce Mann [11] and Troy Bremer [5,*]

Table S1. . Patient clinicopathologic factors, treatment, and events for SweDCIS trial cohort with negative margins and patients included and not included in the Validation Cohort.

	SweDCIS trial Negative margins (*n* = 857)		Complete data subset* (*n* = 582)		SweDCIS trial negative margins (*n* = 857)		Validation Cohort Negative margins (*n* = 504)		SweDCIS trial Negative margins not included in Validation Cohort (*n* = 353)		*p*[*]
**Age, mean (sd)**	**57.1**	**(9.1)**	**57.3**	**(9.1)**			57.6	(9.1)	56.4	(9.0)	0.06
**Year of diagnosis, n (%)**											0.40
1987-1994	491	(57)	329	(57)	491	(57)	295	(59)	196	(56)	
1995-2000	366	(43)	253	(43)	366	(43)	209	(41)	157	(44)	
**Mode of detection, n (%)**											0.16
Screening	689	(80)	464	(80)	689	(81)	414	(82)	275	(78)	
Non screening	165	(19)	117	(20)	165	(19)	89	(18)	76	(22)	
Missing	3	(0)	1	(0)	3	(0)	1	(0)	2	(1)	
**Palpable, n (%)**											0.31
Yes	192	(22)	129	(22)	192	(22)	110	(22)	82	(23)	
No	642	(75)	453	(78)	642	(75)	394	(78)	248	(70)	
Missing	23	(3)	-	-	23	(3)	-	-	23	(7)	
**Size, n (%)**											0.72
≤1 cm	398	(46)	273	(47)	398	(46)	240	(48)	158	(45)	
>1 cm	428	(50)	309	(53)	428	(50)	264	(52)	164	(46)	
Missing	31	(4)	-	-	31	(4)	-	-	31	(9)	
**Nuclear grade, n (%)**											0.38
1	175	(20)	180	(31)	175	(20)	155	(31)	20	(6)	
2	185	(22)	185	(32)	185	(22)	164	(33)	21	(6)	
3	219	(26)	217	(37)	219	(26)	185	(37)	34	(10)	
Missing	278	(32)	-	-	278	(32)	-	-	278	(79)	
**Radiotherapy, n (%)**											0.94
Yes	436	(51)	292	(50)	436	(51)	257	(51)	179	(51)	
No	421	(49)	290	(50)	421	(49)	247	(49)	174	(49)	
**Hormonal therapy, n (%)**											0.85
Yes	30	(4)	19	(3)	30	(4)	17	(3)	13	(4)	
No	827	(96)	563	(97)	827	(96)	487	(97)	340	(96)	

Table S1. (CONTINUED). Patient clinicopathologic factors, treatment, and events for SweDCIS trial cohort with negative margins and patients included and not included in the Validation Cohort.

	**SweDCIS trial Negative margins (*n* = 857)**		**Complete data subset* (*n* = 582)**		**SweDCIS trial negative margins (*n* = 857)**		**Validation Cohort Negative margins (*n* = 504)**		**SweDCIS trial Negative margins not included in Validation Cohort (*n* = 353)**		**p***
**First ipsilateral events within 10-years, n (%)**											0.04
New DCIS	86	(10)	73	(13)	86	(10)	59	(12)	27	(8)	
InvBE - Invasive BC	61	(7)	45	(8)	61	(7)	31	(6)	30	(8)	
InvBE - Metastases	3	(0)	-	-	3	(0)	-	-	3	(1)	
Censored - BC-death	1	(0)	1	(0)	1	(0)	1	(0)	-	-	
Censored - other death	48	(6)	34	(5)	48	(6)	30	(6)	18	(5)	
Censored at end of follow-up	658	(77)	429	(74)	658	(77)	383	(76)	275	(78)	
**First contralateral events within 10 years, n (%)**											0.97
New DCIS	12	(1)	10	(2)	12	(1)	7	(1)	5	(1)	
Invasive BC	34	(4)	25	(4)	34	(4)	21	(4)	13	(4)	

*Comparing the Validation Cohort (*n* = 504) with those with negative margin in SweDCIS but not included in the Validation Cohort. Two-sample t test was used for Age, otherwise Fisher´s exact test was used. Abbreviations: BC = Breast cancer. InvBE = Invasive breast events.

Table S2. Patient clinicopathologic factors, treatment, and events for SweDCIS trial cohort with negative margins included in the Validation Cohort with and without radiotherapy (RT).

	Validation cohort Negative margins (*n* = 504)		Validation Cohort Negative margins No RT (*n* = 247)		Validation Cohort Negative margins, RT (*n* = 257)		p^*
**Age, mean (sd)**	**57.6**	**(9.1)**	57.5	(8.7)	57.7	(9.6)	0.73
**Age, median (min-max)**	57.6	(29-79)	57.3	(29-79)	55.8	(34-76)	0.67
**Age group, n (%)**							0.036
**<50**	116	(23)	56	(23)	60	(23)	
**50-69**	348	(69)	179	(72)	169	(66)	
**≥70**	40	(8)	12	(5)	28	(11)	
**Year of diagnosis, n (%)**							0.59
1987-1994	295	(59)	148	(60)	147	(57)	
1995-2000	209	(41)	99	(40)	110	(43)	
**Mode of detection, n (%)**							0.82
Screening	414	(82)	201	(81)	213	(83)	
Non screening	89	(18)	45	(18)	44	(17)	
Missing	1	(0)	1	(0)	-	-	
**Palpable, n (%)**							0.91
Yes	110	(22)	53	(21)	27	(22)	
No	394	(78)	194	(79)	200	(78)	
**Size, n (%)**							0.93
≤1 cm	240	(48)	117	(47)	123	(48)	
>1 cm	264	(52)	130	(53)	134	(52)	
**Nuclear grade, n (%)**							0.84
1	155	(31)	73	(30)	82	(32)	
2	164	(33)	81	(33)	83	(32)	
3	185	(37)	93	(38)	92	(36)	
**Hormonal therapy, n (%)**							0.81
Yes	17	(3)	9	(4)	8	(3)	
No	487	(97)	238	(96)	249	(97)	

Table S2. (CONTINUED): Patient clinicopathologic factors, treatment, and events for SweDCIS trial cohort with negative margins included in the Validation Cohort without and with radiotherapy (RT).

	Validation Cohort Negative margins (*n* = 504)		Validation Cohort Negative margins No RT (*n* = 247)		Validation Cohort Negative margins, RT (*n* = 257)		p^*
**First ipsilateral events within 10-years, n (%)**							<0.001#
New DCIS	59	(12)	41	(17)	18	(7)	
InvBE - Invasive BC	31	(6)	20	(8)	11	(4)	
InvBE - Metastases	-	-	-	-	-	-	
Censored - BC-death	1	(0)	-	-	1	(0)	
Censored - other death	30	(6)	16	(6)	14	(5)	
Censored at end of follow-up	383	(76)	170	(69)	213	(83)	
**First contralateral events within 10 years, n (%)**							0.071#
New DCIS	7	(1)	5	(2)	2	(1)	
Invasive BC	21	(4)	6	(2)	15	(6)	

[*] Comparing the Validation Cohort (n = 504) with those with negative margin in SweDCIS but not included in the Validation Cohort. Two-sample t test or Mann-Whitney test was used for Age, otherwise Fisher´s exact test was used. [#] When testing differences in number of events, groups are new DCIS, InvBE and censored. Abbreviations: BC = Breast cancer. InvBE = Invasive breast events.

Table S3. Multivariable Cox proportional hazards analysis allowing for interaction between RT and categorial DS, using alternative thresholds for DS > x, in 504 women with complete biosignature data and free margins from the SweDCIS trial.

A. Interaction analysis for 10-year total breast event rates.

Cut-off (DS > x) x	Events and number low risk group (DS ≤ x)	Events and number elevated risk group (DS > x)	RT Effect (No RT/RT) low risk group (DS ≤ x) HR (95% CI), p	RT Effect (No RT/RT) elevated risk group (DS > x) HR (95% CI), p	RT: (DS > x) Interaction LR test p
1.0	22/147	68/357	0.51 (0.22–1.22), *p* = 0.13	**0.38 (0.22–0.63), *p* <.001**	0.52
2.0	29/182	61/322	0.48 (0.22–1.03), *p* = 0.061	**0.37 (0.22–0.64), *p* <.001**	0.57
2.5	30/202	60/302	0.56 (0.27–1.18), *p* = 0.127	**0.34 (0.19–0.58), *p* <.001**	0.26
2.6	30/203	60/301	0.56 (0.26–1.17), *p* = 0.120	**0.34 (0.19–0.59), *p* <.001**	0.28
2.7	33/212	57/292	0.56 (0.28–1.14), *p* = 0.110	**0.33 (0.19–0.58), *p* <.001**	0.23
2.8	37/228	53/276	0.55 (0.28–1.08), *p* = 0.081	**0.32 (0.18–0.58), *p* <.001**	0.23
2.9	39/238	51/266	0.55 (0.28–1.05), *p* = 0.071	**0.32 (0.17–0.58), *p* <.001**	0.22
3.0	40/240	50/264	0.53 (0.28–1.02), *p* = 0.059	**0.32 (0.17–0.58), *p* <.001**	0.24

B. Interaction analysis for 10-year invasive breast event rates

Cut-off (DS > x) x	Events and number low risk group (DS ≤ x)	Events and number elevated risk group (DS > x)	RT Effect (No RT/RT) low risk group (DS ≤ x) HR (95% CI), p	RT Effect (No RT/RT) elevated risk group (DS > x) HR (95% CI), p	RT: (DS > x) Interaction LR test p
1.0	10/147	21/357	0.87 (0.25–3.01), *p* = 0.829	**0.32 (0.13–0.84), *p* = 0.020**	0.21
2.0	12/182	19/322	0.88 (0.28–2.73), *p* = 0.825	**0.29 (0.10–0.79), *p* = 0.016**	0.14
2.5	13/202	18/302	1.10 (0.37–3.28), *p* = 0.860	**0.22 (0.07–0.66), *p* = 0.007**	**0.033**
2.6	13/203	18/301	1.09 (0.37–3.25), *p* = 0.876	**0.21 (0.07–0.64), *p* = 0.006**	**0.035**
2.7	13/212	18/292	1.12 (0.38–3.33), *p* = 0.841	**0.20 (0.07–0.62), *p* = 0.005**	**0.029**
2.8	13/228	18/276	1.15 (0.38–3.41), *p* = 0.807	**0.22 (0.07–0.68), *p* = 0.008**	**0.024**
2.9	14/238	17/266	0.94 (0.33–2.69), *p* = 0.915	**0.22 (0.07–0.69), *p* = 0.009**	0.057
3.0	15/240	16/264	0.84 (0.30–2.31), *p* = 0.734	**0.24 (0.08–0.74), *p* = 0.013**	0.093

Multivariable Cox proportional hazards analysis of the association of RT, categorical DS > x, and their interaction RT:DS > x with 10-year breast event rates. Event counts, patient counts, hazard ratios with 95% confidence intervals and p-values are given in the categorical low (DS ≤ x) and elevated (DS > x) risk groups for continuous DS thresholds between 1.0 and 3.0. The *p*-value for interaction of RT:(DS > x) included in the analysis is also provided. **A.** 10-year total BE risks in the Validation Cohort data; **B.** 10-year invasive BE risks in the Validation Cohort data. The DCISionRT test identified patients in low and elevated risk groups defined as DS ≤ x between x = 1.0 and x = 3.0.

Patients in the low risk groups (DS ≤ x) had non-significant differences in 10-year invasive and total breast event rates. Patients in the elevated risk groups (DS > x) had significant RT reduction on 10-year invasive (HR = 0.20 to HR = 0.32, $p \leq 0.02$) and 10-year total (HR = 0.32 to HR = 0.38, $p < 0.001$) breast event rates. No significant interaction effect of DS risk group and RT was shown for 10-year TotBE, while a significant interaction effect indicated a superior RT response for patients with 2.5 ≤ DS ≤ 2.8 for 10-year InvBE.

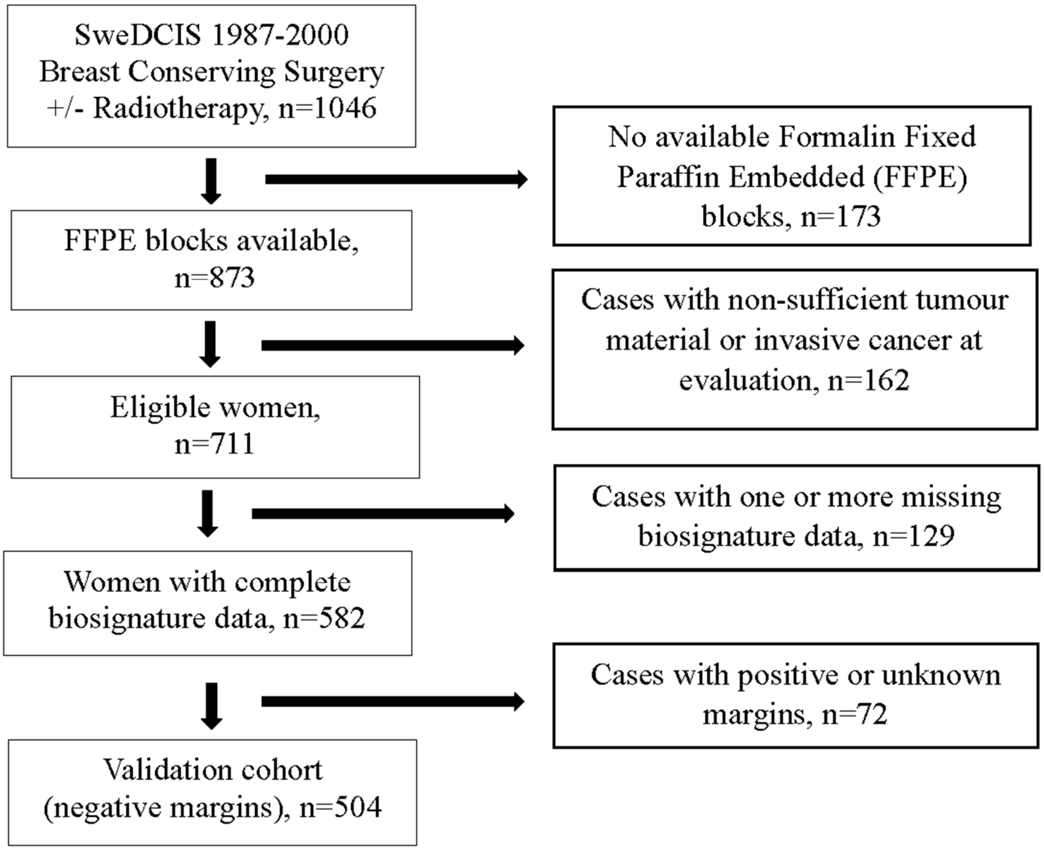

Figure S1. Flow chart describing women from the randomized SweDCIS trial for evaluation of four clinical markers (age, size, surgical margin status, and palpability) and seven biomarkers (PR, HER2, Ki67, FOXA1, COX2, p16/INK4A, SIAH2) included in the DCISionRT biosignature.
